# Optimization of First‐Line Treatment Options in HER2‐Altered Lung Adenocarcinoma: A Real‐World Study

**DOI:** 10.1002/cam4.71260

**Published:** 2025-09-17

**Authors:** Xiufen Wang, Dahai Wang, Yanxin Sun, Yiling Gan, Juan Li, Xuebing Fu, Yihui Ge, Shuyun Wang, Leirong Wang, Haodong Sun, Haifeng Sun, Yuping Sun, Aiqin Gao

**Affiliations:** ^1^ Phase I Clinical Research Center, Shandong Cancer Hospital and Institute, Shandong First Medical University, Shandong Academy of Medical Sciences Jinan Shandong China; ^2^ Department of Thoracic Radiation Oncology Shandong Cancer Hospital and Institute, Shandong First Medical University, Shandong Academy of Medical Sciences Jinan Shandong China; ^3^ Cheeloo College of Medicine, Shandong University Jinan Shandong China; ^4^ School of Clinical Medicine Shandong Second Medical University Weifang Shandong China

**Keywords:** beneficiary populations, first‐line treatment, HER2, immunotherapy, non‐small cell lung cancer

## Abstract

**Objectives:**

HER2 alterations are identified in 2%–4% of lung adenocarcinoma, indicating poor clinical outcomes. Chemotherapy alone (C) or in combination with bevacizumab (BC), immune checkpoint inhibitors (IC), or both (IBC) are standard options in the first‐line setting; however, optimal therapy and beneficiary populations remain unclear.

**Methods:**

Patients with Stage IV HER2‐altered lung adenocarcinoma from four cancer centers in China were retrospectively analyzed. The patients received C, BC, IC, or IBC as the first‐line treatments. Clinical outcomes, including progression‐free survival (PFS) and overall survival (OS), were evaluated. The tumor immune microenvironment (TIME) characteristics were analyzed to explore the beneficiary populations with different treatments.

**Results:**

IBC treatment generated the longest mPFS and OS among four schemes. Subgroup analyses showed that IBC resulted in longer PFS in patients with brain or bone metastases compared to IC. IBC generated significant benefits in younger patients, nonsmokers, and those with oligometastases versus BC. In patients with low density of PD‐1^+^CD8^+^ T‐cells or high density of CD163^+^ macrophages in the TIME, IBC treatment resulted in the longest PFS, followed by BC treatment.

**Conclusions:**

IBC treatment generated optimal efficacy as first‐line therapy for HER2‐altered lung adenocarcinoma. Patients with low PD1^+^CD8^+^ T‐cell or high CD163^+^ macrophage infiltration benefited more from IBC treatment.

## Graphical abstract

Chemotherapy combination with bevacizumab and immune checkpoint inhibitors (IBC) generated optimal efficacy as first‐line therapy for HER2‐altered lung adenocarcinoma. Patients with low PD1^+^CD8^+^ T‐cell or high CD163^+^ macrophage infiltration benefited more from IBC treatment.

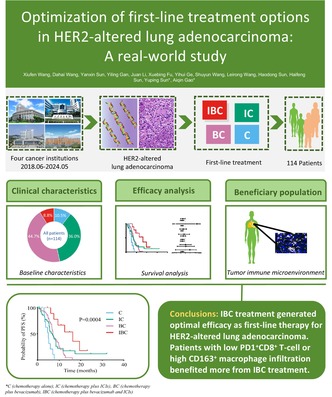

## 1 Introduction

HER2 alteration is a rare but widely acknowledged oncogenic driver of non‐small cell lung cancer (NSCLC), which includes HER2 overexpression, amplification, and gene mutation with incidence rates of 7.7%–23%, 2%–22%, and 1%–6.7%, respectively [1]. HER2 alterations are mainly found in women, non‐smokers, and patients with adenocarcinoma [2], predicting advanced stages, central nervous system metastases, and poor survival [3, 4]. HER2‐altered NSCLC have a mPFS of merely 4.9–5.9 months and median overall survival (mOS) of 9.9–10.7 months [5, 6].

In recent years, targeted therapy against HER2 has been identified as an important strategy for HER2‐altered NSCLC and has achieved major breakthroughs. The DESTINY‐Lung 01 and DESTINY‐Lung 02 studies showed that DS‐8201 resulted in a mPFS of 8.2 months and a mOS of 17.8 months in the later‐line treatment of HER2‐mutated NSCLC and was subsequently approved by the US Food and Drug Administration as a standard treatment option [7, 8]. However, no clinical study has evaluated the first‐line benefit of targeted therapy, including DS‐8201, other anti‐HER2 agents, small‐molecule tyrosine kinase inhibitors (TKIs), or antibody‐conjugating drugs for HER2‐altered NSCLC. Therefore, the first‐line treatment options for HER2‐altered lung adenocarcinoma remain the same as those for oncogene wild‐type patients, using platinum‐based doublet chemotherapy in combination with immune checkpoint inhibitors (ICIs) and/or bevacizumab. However, the efficacy of these treatments for HER2‐altered NSCLC is far from satisfactory.

Bevacizumab in combination with chemotherapy prolongs first‐line PFS compared with chemotherapy alone in HER2‐altered lung adenocarcinoma [9]. Whereas ICIs alone or in combination with platinum‐doublet chemotherapy do not improve PFS and OS compared with chemotherapy in several real‐world studies [10, 11]. This is explained by the fact that HER2‐altered lung adenocarcinoma has a much lower programmed death ligand‐1 (PD‐L1) and tumor mutational burden (TMB) expression than other mutations, leading to a suppressive tumor immune microenvironment (TIME) and hyporesponsiveness to immunotherapy in these patients [12, 13]. Consequently, the combination of bevacizumab and ICIs for the treatment of lung adenocarcinoma has recently gained interest because bevacizumab can reprogram the suppressive TIME toward an immunosupportive state and improve ICI efficacy [14]. The combination of bevacizumab with ICIs and platinum‐containing chemotherapy (ABCP) has shown substantial survival benefits in patients with metastatic NSCLC with active EGFR mutations who are resistant to EGFR‐TKIs (a population that was previously considered unfriendly to immunotherapy) compared with BCP treatment [15], verifying their synergistic effect. However, whether ABCP improves survival in patients with HER2‐altered NSCLC remains unclear.

Therefore, we aimed to analyze the efficacies of different first‐line treatment options for HER2‐altered lung adenocarcinoma using real‐world data and identify the target population that could benefit from the different treatments.

## 2 Materials & Methods

### 2.1 Inclusion Criteria

Patients with HER2‐altered advanced NSCLC were retrospectively enrolled from four cancer institutions in China (Shandong Cancer Hospital, Shandong Provincial Hospital, Affiliated Hospital of Qingdao University and Yantai Yuhuanding Hospital) between June 1, 2018 and May 15, 2024. The inclusion criteria were as follows: (1) pathologically confirmed diagnosis of NSCLC; (2) Stage IV disease according to the American Joint Committee on Cancer Staging Manual version 8; (3) confirmation of HER2 gene mutation by FISH, NGS, or PCR; (4) patients receiving chemotherapy alone (C) or chemotherapy combinations (including chemotherapy plus ICIs (IC), chemotherapy plus bevacizumab (BC), or chemotherapy plus bevacizumab and ICIs (IBC)) as first‐line treatment.

### 2.2 Data Collection and Assessment

Clinicopathological characteristics and molecular information, including age, gender, smoking status, body mass index (BMI), Eastern Cooperative Oncology Group (ECOG) score, site and number of metastases, type of HER2 alteration, PD‐L1 expression, treatment regimen and duration, and adverse events, were recorded.

Tumor response and progression were evaluated according to the Solid Tumors Response Evaluation Criteria version 1.1 and classified as complete response (CR), partial response (PR), stable disease (SD), or progressive disease (PD). The percentage of patients who achieved CR and PR was defined as the objective response rate (ORR). The sum of the CR, PR, and SD rates was defined as the disease control rate (DCR). The period from the start of the first‐line treatment to the date of disease progression or death from any cause was used to calculate PFS. The period from the first‐line treatment to the date of death from any cause was used to calculate OS. The last follow‐up visit was on October 17, 2024. Adverse events (AEs) were evaluated according to the Common Terminology Criteria for Adverse Events, v5.0.

### 2.3 Multiplex Immunofluorescence (mIF) Assays

Paraffin‐embedded tissue sections were collected from patients before first‐line treatment, and the percentages of immune cell subsets, including CD4^+^, CD8^+^, FOXP3^+^, granzyme B (GZMB)^+^, PD‐1^+^, CD68^+^, and CD163^+^ cells, were detected by mIF. mIF staining was performed using a 7‐color fluorescence immunohistochemistry kit (10,268,100,010; Panovue, China). Briefly, sections (4 μm thick) were obtained from formalin‐fixed paraffin‐embedded lung cancer tissues. After deparaffinization and rehydration in a descending ethanol series, Tris‐EDTA buffer (pH 9) or citrate buffer (PH 6) was used for antigen retrieval in a microwave oven. After protein blocking with 1% bovine serum albumin, mIF staining was performed sequentially for the primary antibody and the corresponding secondary horseradish peroxidase‐conjugated antibody against mouse or rabbit immunoglobulins (Absin, Shanghai, China). The primary antibodies used were as follows: Pan‐cytokeratin (1:500, Abcam, ab7753), GZMB (1:200, Abcam, ab255598), CD4 (1:2000, Abcam, ab183685), Foxp3 (1:500, Abcam, ab215206), PD1 (1:200, Abcam, ab237728), CD8 (1:400, CST, 85336), CD163 (1:500, CST, 93498), and CD68 (1:400, CST, 76437). The slides were then incubated with different opal fluorophores (1:100) diluted in 1 × Plus Amplification Diluent (Absin, Shanghai, China). All fluorescently labeled slides were scanned using an OLYMPUS VS200 microscope (Olympus, Tokyo, Japan) at 40 × magnification with appropriate exposure times, and five representative regions were randomly selected. The percentage of different cell subsets was determined as the ratio of positively stained cells to all nucleated cells. All images were independently reviewed by two professional senior pathologists before data were exported, and at least 20% of each visual field was reviewed.

The frequencies of the following cell subsets were calculated: CD4^+^, CD8^+^, CD4^+^FOXP3^+^(Tregs), PD1^+^CD8^+^ (exhausted CD8^+^T cells), GZMB^+^CD8^+^ (cytotoxic CD8^+^T cells), CD68^+^, and CD163^+^CD68^+^ (M2‐like macrophages).

### 2.4 Statistical Analysis

SPSS version 26.0 and GraphPad Prism 8 software were used for the statistical analyses. Differences in the ORR and DCR between the two groups were calculated using the chi‐square test or Fisher's exact test. Survival was summarized and analyzed using the Kaplan–Meier method and log‐rank test. Subgroup analyses were performed using Cox proportional hazards regression models. Cox proportional hazards regression models were used to assess the hazard ratios (HRs) and corresponding 95% confidence intervals (95% CI). *p* < 0.05 was considered statistically significant.

## 3 Results

### 3.1 Clinical Characteristics

A total of 114 patients with HER2 mutations (99/114), amplification (11/114), or both (4/114) were included, of whom 12 received C, 41 received BC, 51 received IC, and 10 received IBC treatment. The median age of all patients was 60.5 years (range: 26–78 years). Overall, 43.9% of the patients were women and 61.4% were nonsmokers. The baseline characteristics of the four groups were largely balanced. A detailed summary of the results is presented in Table 1.

**TABLE 1:** Baseline characteristics.

Variables	All patients (*n* = 114) (%)	C (*n* = 12)	IC (*n* = 41)	BC (*n* = 51)	IBC (*n* = 10)	*p* ^a^
Age (y), median (range)	60.5 (26–78)	56 (44–69)	61 (26–77)	60 (32–76)	59.5 (48–78)	0.363
Gender (%)						
Female	50 (43.9)	5 (46.7)	15 (45.8)	26 (51.0)	4 (40.0)	0.578
Male	64 (56.1)	7 (58.3)	26 (63.4)	25 (49.0)	6 (50.0)	
Smoking (%)						
Never	70 (61.4)	6 (50.0)	24 (58.5)	34 (66.7)	6 (60.0)	0.696
Ever	44 (38.6)	6 (50.0)	17 (41.5)	17 (33.3)	4 (40.0)	
BMI						
< 18.5	2 (1.8)	1 (8.3)	1 (2.4)	0 (0)	0 (0)	0.651
18.5–23.9	50 (43.8)	7 (58.3)	19 (46.4)	20 (39.2)	4 (40)	
≥ 23.9	38 (33.3)	3 (25)	16 (39)	16 (31.4)	3 (30)	
NA	24 (21.1)	1 (8.4)	5 (12.2)	15 (29.4)	3 (30)	
ECOG						
0	3 (2.6)	0 (0)	1 (2.4)	1 (2.0)	1 (10)	0.207
1	44 (38.6)	3 (25)	20 (48.8)	18 (35.3)	3 (30)	
2	43 (37.7)	8 (66.7)	15 (36.6)	17 (33.3)	3 (30)	
NA	24 (21.1)	1 (8.3)	5 (12.2)	15 (29.4)	3 (30)	
Distant metastasis no.(M ≥ 3) (%)	26 (22.8)	1 (8.3)	7 (17.1)	16 (31.4)	2 (20.0)	0.266
Brain metastasis	33 (28.9)	1 (8.3)	16 (39.0)	12 (23.5)	4 (40.0)	0.118
Bone metastasis	54 (47.4)	3 (25.0)	14 (34.1)	32 (62.7)	5 (50.0)	0.016
HER2 mutation type						
Mutation	99 (86.8)	10 (83.3)	34 (82.9)	45 (88.2)	10 (100.0)	0.782
Amplification	11 (9.6)	1 (8.3)	5 (12.2)	5 (9.8)	0 (0.0)	
Both	4 (3.5)	1 (8.3)	2 (4.9)	1 (2.0)	0 (0.0)	
PD‐L1expression						
< 1%	39 (34.2)	1 (8.3)	13 (31.7)	22 (43.1)	3 (30.0)	0.22
≥ 1%	28 (17.5)	2 (16.7)	13 (21.9)	9 (17.6)	4 (0.0)	
NA	47 (41.2)	9 (75.0)	15 (36.6)	20 (39.2)	3 (30.0)	

Abbreviations: BMI, body mass index (BMI = weight/height squared) (kg/m^2^); ECOG, Eastern Cooperative Oncology Group; NA, not applicable; PD‐L1, programmed death‐ligand 1.

^a^ Categorical and continuous variables were compared using chi‐squared and Kruskal–Wallis tests, respectively.

### 3.2 Efficacy of Different Treatments

**FIGURE 1:**
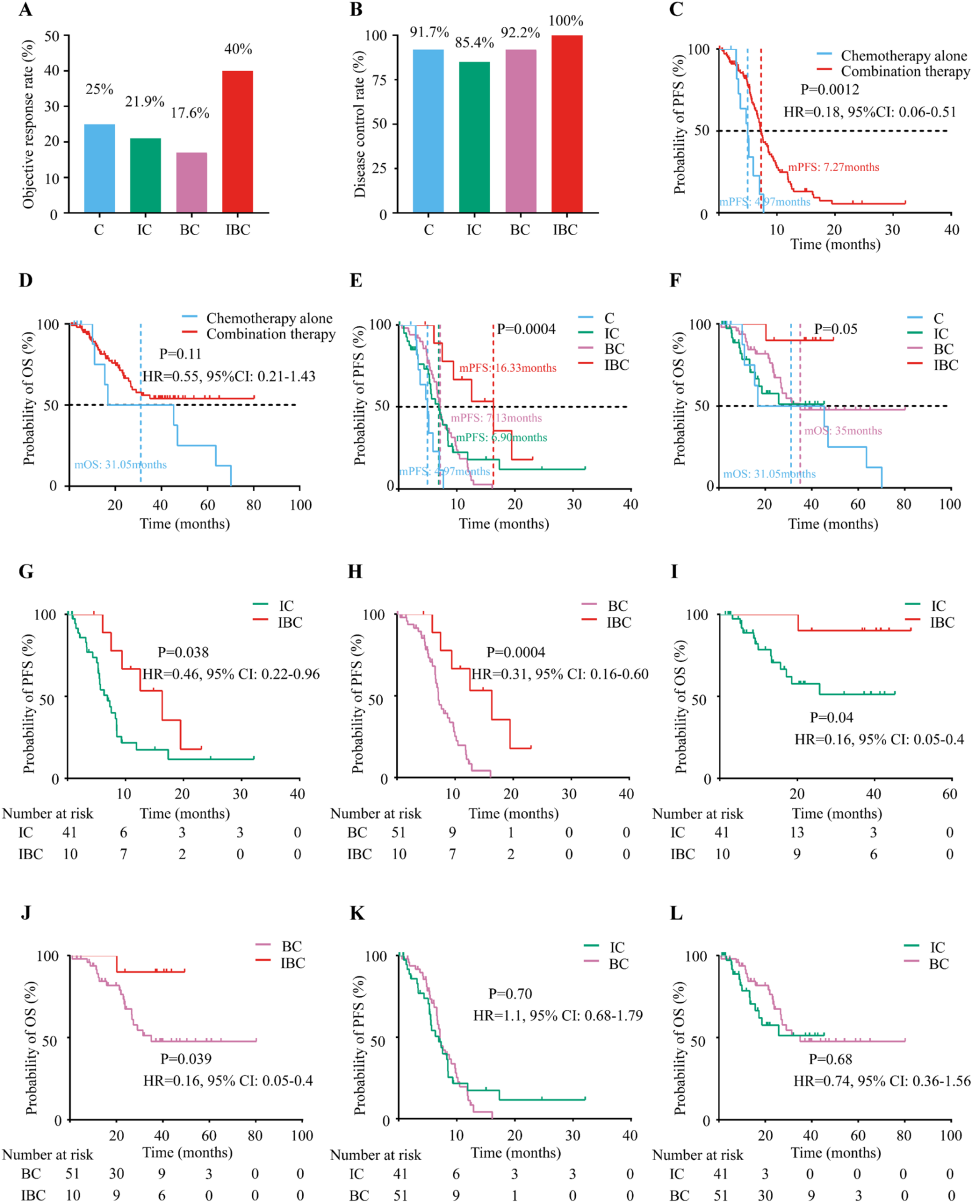
Objective response rate, disease control rate, progression‐free survival, and overall survival in enrolled patients.

The ORR and DCR for all patients was 21.9% and 90.3%, respectively. The IBC treatment group had the highest ORR among the four groups (40%, IBC group; 25%, C group; 21.9%, IC group; and 17.6% BC group; Figure 1A). However, the DCR in the four groups was comparable (100%, IBC group; 91.7%, C group; 85.4%, IC group; and 92.2%, BC group; Figure 1B). Detailed information is provided in Table 2.

**TABLE 2:** Efficacy of different treatments.

Efficacy *n* (%)	All patients (*n* = 114)	C (*n* = 12)	IC (*n* = 41)	BC (*n* = 51)	IBC (*n* = 10)	*p* ^a^
PR	25 (21.9)	3 (25.0)	9 (21.9)	9 (17.6)	4 (40.0)	
SD	78 (68.4)	8 (66.7)	26 (63.4)	38 (74.5)	6 (60.0)	
PD	11 (9.6)	1 (8.3)	6 (14.6)	4 (7.8)	0 (0.0)	
ORR	25 (21.9)	3 (25.0)	9 (21.9)	9 (17.6)	4 (40.0)	0.47
			9 (21.9)	9 (17.6)		0.61
			9 (21.9)		4 (40.0)	0.25
				9 (17.6)	4 (40.0)	0.20
DCR	103 (90.3)	11 (91.7)	35 (85.4)	47 (92.2)	10 (100.0)	0.14
		—	35 (85.4)	47 (92.2)	—	0.57
		—	35 (85.4)	—	10 (100.0)	0.18
		—	—	47 (92.2)	10 (100.0)	0.99

Abbreviations: DCR, disease control rate; ORR, objective response rate; PD, progressive disease; PR, partial response; SD, stable disease.

^a^ Categorical and continuous variables were compared using chi‐squared and Kruskal–Wallis tests, respectively.

Patients who received chemotherapy‐based combination therapy had superior mPFS and numerically longer mOS relative to those receiving chemotherapy alone (PFS: 7.27 months vs. 4.97 months, *p* = 0.0012; OS: not arrival (NA) vs. 31.05 months, *p* = 0.11; Figure 1C,D). Further analysis showed that the combination treatment resulted in a better PFS than chemotherapy alone (Figure S1A–C); IBC had the longest PFS among the four groups (*p* = 0.0004) (Figure 1E). Similarly, IBC resulted in a significantly better OS than chemotherapy alone, IC, and BC (*p* = 0.03) (Figure 1F; Figure S1D–F). Subsequently, we performed pairwise comparisons of the combination treatments. The results showed that compared with IC (mPFS: 16.33 months vs. 6.90 months; HR = 0.46; *p* = 0.038; Figure 1G) or BC (mPFS: 16.33 months vs. 7.13 months; HR = 0.31; p = 0.0004; Figure 1H), treatment with IBC resulted in superior PFS. Meanwhile, OS was significantly longer in the IBC group than that in the IC (mOS: NA vs. NA; HR = 0.16; *p* = 0.04; Figure 1I) and BC groups (mOS: NA vs. 35.0 months; HR = 0.016; *p* = 0.039; Figure 1J). However, no difference was observed in PFS (HR = 1.1, 95% CI: 0.68–1.79, Figure 1K) and OS (HR = 0.74, 95% CI: 0.36–1.56, Figure 1L) between the BC and IC groups. These results suggest that IBC treatment should be preferred in the first‐line treatment of HER2‐altered NSCLC.

**FIGURE 2:**
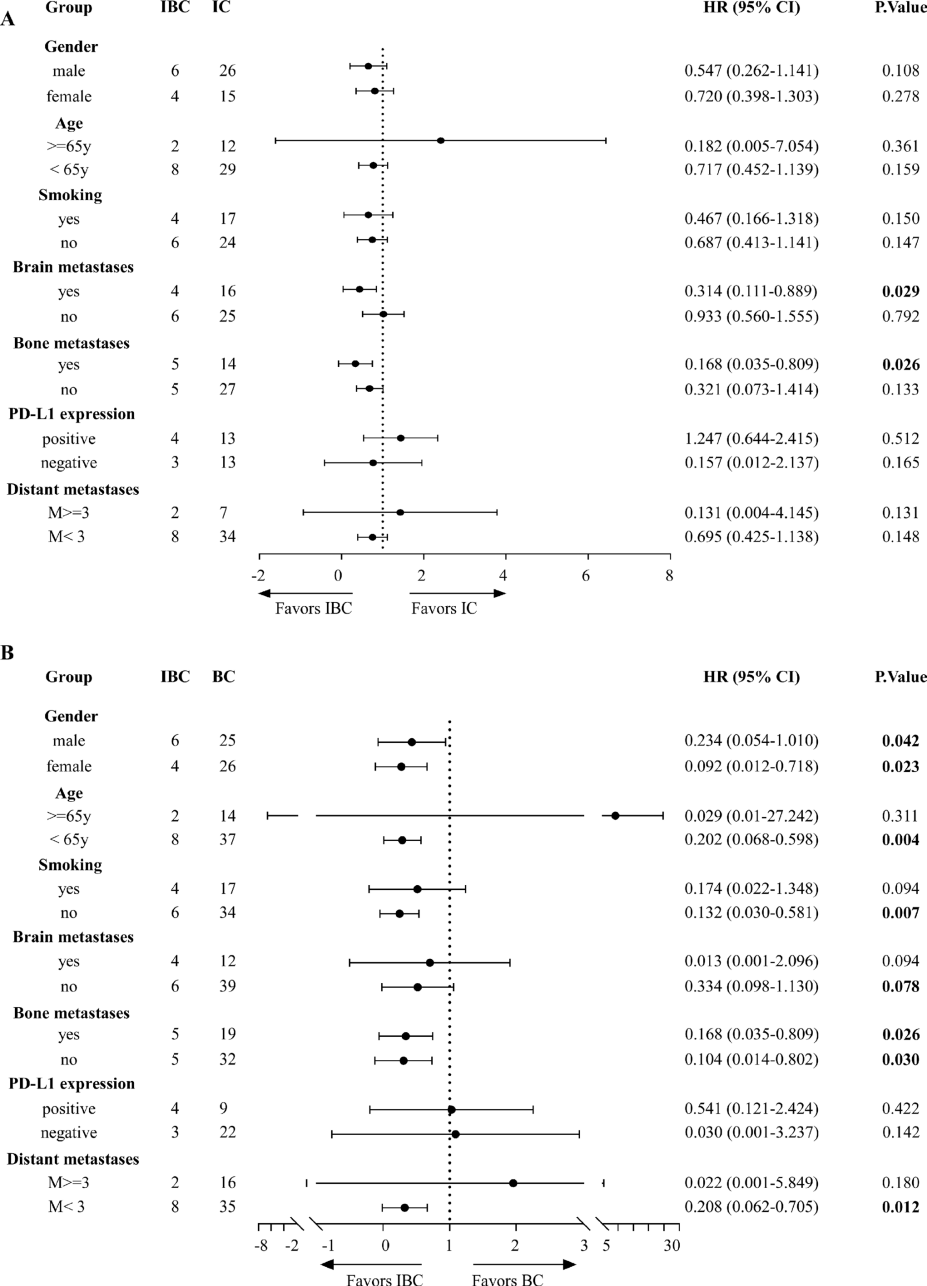
Forest plot of subgroup analyses of progression‐free survival in enrolled patients.

**FIGURE 3:**
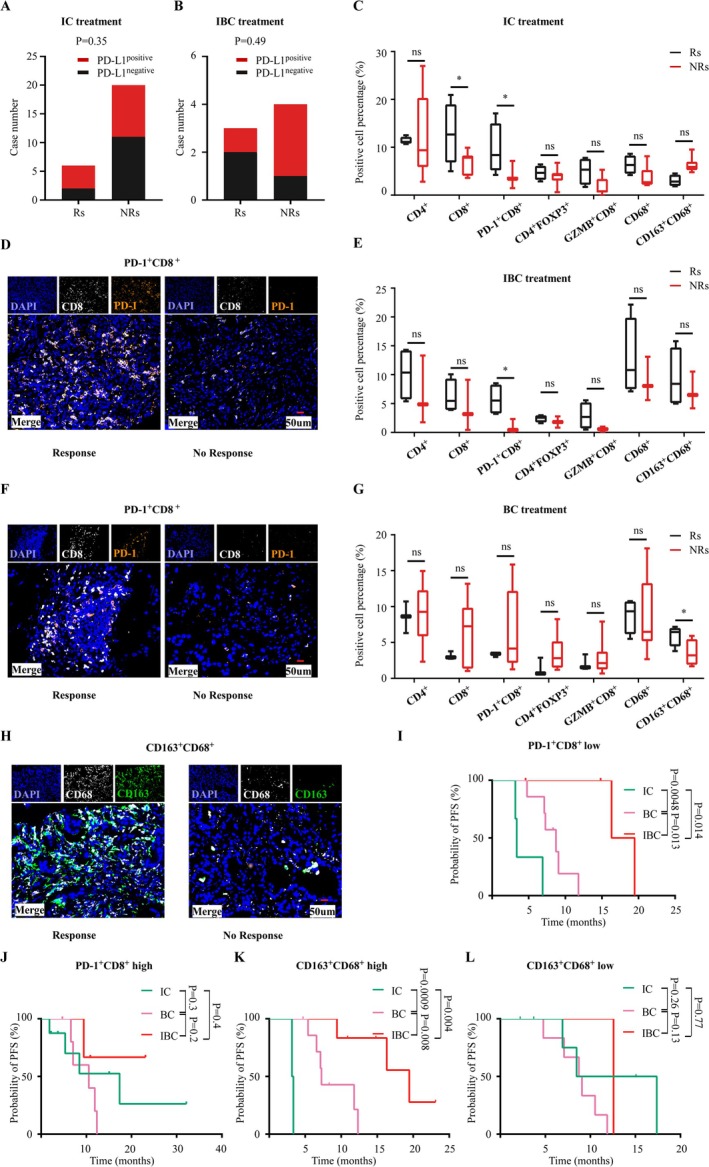
Correlations between the TIME characteristics and treatment efficacy in different schemes. (A, B) Correlations between PD‐L1 expression and treatment response in patients treated with IC or IBC treatments; (C–H) Correlations between different T cell and TAM subset infiltration and treatment response in three treatment groups; (I, J) PFS upon three treatment schemes in patients with high or low PD1^+^CD8^+^ T‐cell infiltration; (K, L) PFS upon different schemes in patients with high or low CD163^+^CD68^+^ TAM infiltration. R, patients achieved complete response or partial response; NR, patients achieved stable disease or disease progression; ns, no significance. Positive cell percentage = (number of positive cells/total number of nucleated cells) *100%. Plotting scale: 50 μm (two‐sided unpaired t‐test, ns not significant, *p < 0.05).

We also performed a subgroup analysis of PFS based on clinical characteristics (Figure 2). IBC showed a more significant PFS benefit in patients with brain and bone metastases compared with IC (Figure 2A). In addition, IBC resulted in better PFS in nonsmokers and patients with younger ages (< 65 years) and oligometastasis than BC (Figure 2B). Unfortunately, we did not observe a difference in PFS among the three groups based on PD‐L1 expression, which is a crucial positive biomarker of immunotherapy efficacy (Figure 3A,B). Moreover, the IC and BC showed no survival differences in either subgroup (Figure S2). These results suggest that IBC should be preferably selected for younger patients, or those with brain metastasis, bone metastasis, and oligometastasis, and that PD‐L1 level is not an effective biomarker for IBC treatment.

### 3.3 Beneficiary Population Based on Immune Microenvironment Analyses

To accurately screen out patients who can benefit from the different treatment regimens, we classified patients into responders (Rs) and non‐responders (NRs) and analyzed TIME features, including PD‐L1 expression and densities of different T cell and tumor‐associated macrophage (TAM) subsets, two major immunocyte components in the TME [16]. As expected, we did not observe any difference in PD‐L1 expression between the Rs and NRs who received IC or IBC regimens (Figure 3A,B). However, we found that Rs to IC and IBC regimens had a significantly higher PD1^+^CD8^+^ T‐cell frequency compared with NRs (Figure 3C–F), while Rs to the BC regimen showed higher levels of CD163^+^ TAM infiltration (Figure 3G,H). These results suggest that PD1^+^CD8^+^ T‐cell and CD163^+^ TAM infiltration may affect the efficacy of combination therapies in HER2‐altered NSCLC.

Subsequently, we explored whether the levels of PD1^+^CD8^+^ T‐cells and CD163^+^ TAM infiltration in the TME could be used to optimize treatment choices for HER2‐altered NSCLC. Patients were stratified into high and low groups based on the median PD1^+^CD8^+^ T‐cell or CD163^+^ TAM frequencies, and PFS in the IBC, IC, and BC groups was compared. Patients with low PD‐1^+^CD8^+^ T‐cell infiltration in the TME exhibited the longest PFS with IBC, followed by BC, while IC had the worst PFS (Figure 3I). However, in patients with high PD1^+^CD8^+^ T‐cell density, IBC, IC, and BC showed comparable PFS (Figure 3J). Similarly, in patients with high levels of CD163^+^ TAM, IBC exhibited the longest PFS, followed by BC and IC (Figure 3K). However, in patients with low CD163^+^ TAM infiltration, no difference in PFS was observed between the three treatments (Figure 3L). These results indicate that patients with low PD1^+^CD8^+^ T‐cell and high CD163^+^ TAM infiltration in the TME should be preferably administered IBC, whereas IBC is not necessary for the remaining patients because of higher economic and physiological toxicity.

### 3.4 Safety

AEs related to treatment were assessed and graded according to the CTCAE 5.0 classification. In general, the incidence of adverse effects in the C, IC, BC, and IBC groups was 25.0%, 31.4%, 21.6%, and 40.0%, respectively, and no significant difference in AEs of the four treatments was found (*p* = 0.461). Gastrointestinal toxicity was the most frequent grade I–II adverse event in the C (16.7%), BC (7.8%), and IBC treatment groups (30.0%), whereas myelosuppression was the most frequent event in the IC (14.6%) group. Additionally, myelosuppression was the predominant grade III–IV adverse event across the three groups. Moreover, IBC treatment led to hepatotoxicity (20%), which was higher than that observed in the IC (2.4%) and BC (2.0%) groups. However, thyroid toxicity was only observed in the IBC group, while pneumonia, cardiotoxicity, hypokalemia, phlebothrombosis, and proteinuria were observed only in the IC group. These results suggest that the safety of the IBC treatment is acceptable. All adverse events are presented in Table 3.

**TABLE 3:** Adverse events based on different treatment options.

Adverse events	C (*n* = 12)	IC (*n* = 41)	BC (*n* = 51)	IBC (*n* = 10)
Any grade (person‐time)	3 (25.0%)	14 (34.1%)	11 (21.6%)	4 (40%)

	Grades 1–2	Grades 3–4	Grades 1–2	Grades 3–4	Grades 1–2	Grades 3–4	Grades 1–2	Grades 3–4
Gastrointestinal toxicity	**2 (16.7)**	**—**	**3 (7.3)**	**—**	**4 (7.8)**	**—**	**3 (30.0)**	**—**
Myelosuppression			**6 (14.6)**	**4 (9.8)**	**4 (7.8)**	**2 (3.9)**	—	**1 (10.0)**
Leukopenia	—	—	4 (9.8)	2 (4.9)	3 (5.9)	1 (2.0)	—	1 (10.0)
Neutropenia	—	—	2 (4.9)	1 (2.4)	1 (2.0)	1 (2.0)	—	—
Thrombocytopenia	1 (8.3)	—	—	1 (2.4)	—	—	—	—
Hepatotoxicity	—	—	**1 (2.4)**	—	—	**1 (2.0)**	**1 (10.0)**	**1 (10.0)**
Thyroid toxicity	—	—	—	—	—	—	**2 (20.0)**	—
Pneumonia	—	—	—	**1 (2.4)**	—	—	—	—
Cardiotoxicity	—	—	**1 (2.4)**	**—**	**—**	**—**	**—**	**—**
Hypokalemia	**—**	**—**	**—**	**1 (2.4)**	**—**	**—**	**—**	**—**
Phlebothrombosis	**—**	**—**	**1 (2.4)**	**—**	**—**	**—**	**—**	**—**
Proteinuria	**—**	**—**	**1 (2.4)**	**—**	**—**	**—**	**—**	**—**

*Note:* The bolded portion represents the sum total of the adverse reaction data for this category.

## 4 Discussion

This study analyzed the efficacies of different first‐line treatment options for HER2‐altered lung adenocarcinoma and identified the target population that could benefit from the different treatments. Using real‐world data, we found that the IBC generated optimal efficacy as a first‐line therapy for HER2‐altered NSCLC, which was consistent in most subgroups. Additionally, in our effort to explore biomarkers for beneficiary population screening, we found that patients with low PD1^+^CD8^+^ T‐cell or high CD163^+^ macrophage infiltration benefit more from IBC treatment.

The combination of bevacizumab and ICIs has shown promising efficacy in the treatment of NSCLC, particularly in patients with active EGFR mutations [17]. However, whether this combination generates a synergistic effect in HER2‐altered lung adenocarcinomas was unknown. To the best of our knowledge, this is the first study to demonstrate the PFS and OS benefit of IBC treatment versus other combination therapies in patients with HER2‐altered lung adenocarcinomas. Additionally, this advantage was consistent in most subgroups, including those with brain, bone, and oligometastases. Moreover, IBC did not significantly increase the AEs. Thus, our findings suggest that IBC should be prioritized as a first‐line therapy for HER2‐altered lung adenocarcinoma. Mechanistically, bevacizumab‐induced normalization of the tumor vascular network can directly alleviate hypoxia, increase tumoricidal T‐cell infiltration, and prevent the recruitment of inhibitory regulatory T cells and myeloid‐derived suppressor cells [14, 18, 19]. In addition, bevacizumab induces dendritic cell differentiation and maturation, polarizes TAMs to an antitumor M1‐like phenotype, and enhances intrinsic anti‐tumor immunity [20]. These mechanisms may explain the findings of the present study. However, the clinical synergy between ICIs and bevacizumab in HER2‐altered lung adenocarcinoma requires further verification in prospective Phase III clinical trials.

Furthermore, we analyzed the correlation between tumor response and TIME characteristics, including PD‐L1 expression levels, to identify predictive biomarkers for first‐line treatment efficacy and to screen the subpopulation that could benefit from these treatments. Unfortunately, PD‐L1 expression, the most commonly used biomarker for ICI efficacy, showed no correlation with tumor response, even in the IC group with HER2‐altered lung adenocarcinoma. Therefore, we further analyzed the TIME characteristics to identify predictive biomarkers of first‐line treatment efficacy. We found that compared to NRs, Rs to IC treatment showed much higher numbers of PD‐1^+^CD8^+^ T‐cells in the TIME, while Rs to BC showed higher levels of CD163^+^ TAMs. Although T cells and macrophages are the most abundant immune cells in the TME, CD8^+^ T‐cells are the main cell population of the immune system that kill tumors. The key feature of CD8^+^ T cell depletion is the simultaneous and sustained upregulation of multiple immunosuppressive receptors on the cell surface, among which PD‐1 is the most critical [21, 22]. Thus, PD1^+^CD8^+^ TILs have been reported to be superior in predicting ICI efficacy [21]. Moreover, CD163 is a well‐recognized marker of M2‐like TAMs. Polarization of TAMs toward M2‐like phenotypes is accompanied by their pro‐tumoral function. M2‐like TAMs not only promote tumor angiogenesis through the expression of hypoxia‐associated genes and the secretion of various cytokines [23, 24], but also induce immune suppression by impairing T‐cell immunity [25]. Thus, high infiltration of M2‐like TAMs is associated with poor prognosis [26]. Consequently, our findings highlight PD1^+^CD8^+^ T‐cells and M2‐like TAMs as potential predictive biomarkers for different first‐line treatments.

Notably, we found that these biomarkers can be used to guide first‐line decision‐making. For example, IBC should be preferred for patients with low PD1^+^CD8^+^ T‐cell or high CD163^+^ TAM infiltration in the TME because low PD1^+^CD8^+^ T‐cell and high CD163^+^ TAM represent two major immunosuppressive phenotypes in HER2‐altered lung adenocarcinoma. The addition of bevacizumab should reprogram the immunosuppressive TME toward immune activation, thereby improving ICI efficacy [27, 28]. In contrast, T cells can be easily activated in patients with high PD1^+^CD8^+^ T‐cell or low CD163^+^ TAM infiltration by ICI alone or in combination with chemotherapy; therefore, the addition of bevacizumab is not necessary due to similar efficacy, but higher economic and physiological toxicity. Collectively, our results identified novel biomarkers for different treatments, which may be helpful in accurately screening beneficiary populations.

## 5 Conclusion

IBC treatment generated optimal efficacy as first‐line therapy for HER2‐altered lung adenocarcinoma, which was consistent in most subgroups. Patients with low PD1^+^CD8^+^ T‐cell or high CD163^+^ macrophage infiltration benefited more from IBC treatment. Our findings provide clinical evidence for optimizing first‐line treatment for HER2‐altered lung adenocarcinoma.

## Author Contributions

**Xiufen Wang:** conceptualization (equal), data curation (equal), methodology (equal), software (equal), writing – original draft (lead). **Dahai Wang:** data curation (equal), investigation (equal), methodology (equal), software (equal). **Yanxin Sun:** data curation (equal), formal analysis (equal), methodology (equal), software (equal). **Yiling Gan:** data curation (equal), investigation (equal), resources (equal), validation (equal). **Juan Li:** conceptualization (equal), formal analysis (equal), funding acquisition (equal), supervision (equal). **Xuebing Fu:** formal analysis (equal), project administration (equal), software (equal). **Yihui Ge:** investigation (equal), project administration (equal), supervision (equal). **Shuyun Wang:** project administration (equal), supervision (equal), visualization (equal). **Leirong Wang:** project administration (equal), supervision (equal), visualization (equal). **Haodong Sun:** investigation (equal), software (equal). **Haifeng Sun:** data curation (equal), validation (equal). **Yuping Sun:** conceptualization (equal), formal analysis (equal), funding acquisition (equal), project administration (equal), supervision (equal). **Aiqin Gao:** conceptualization (equal), funding acquisition (equal), project administration (equal), resources (equal), writing – review and editing (equal).

## Ethics Statement

This study was approved by the Shandong Cancer Hospital and Institute review board (SDTHEC202412006), and all patients provided written informed consent. The study was conducted in compliance with the Declaration of Helsinki.

## Consent

The authors have nothing to report.

## Conflicts of Interest

The authors declare no conflicts of interest.

## Supporting information


**Figure S1:** Progression‐free survival in enrolled patients.

**Figure S2:** Forest plot of subgroup analyses of progression‐free survival in enrolled patients.

## Data Availability

The data are not publicly available due to [restrictions e.g., their containing information that could compromise the privacy of research participants].
