# Supplementary figures and images for: Optimization of First‐Line Treatment Options in HER2‐Altered Lung Adenocarcinoma: A Real‐World Study
Source: Cancer Med. 2025 Sep 17;14(18):e71260. doi: 10.1002/cam4.71260 (PMC12441807; doi:10.1002/cam4.71260)

**Supplementary Information**


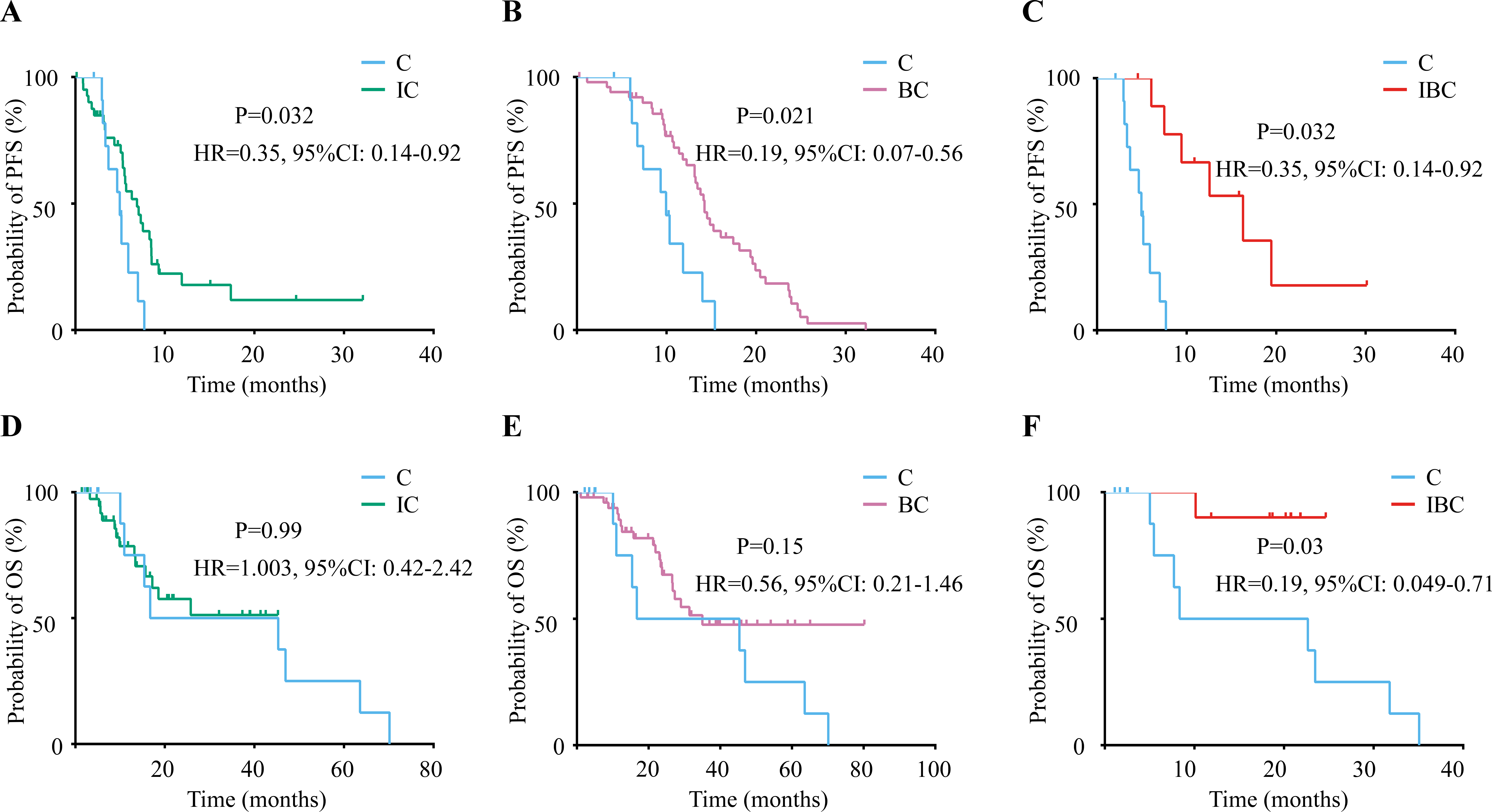


**Figure s1** Progression-free survival in enrolled patients.

Supplement: Supplementary file 1 — Figure S1: Progression‐free survival in enrolled patients. [file CAM4-14-e71260-s002.docx]
