# Supplementary material for: Optimization of First‐Line Treatment Options in HER2‐Altered Lung Adenocarcinoma: A Real‐World Study
Source: Cancer Med. 2025 Sep 17;14(18):e71260. doi: 10.1002/cam4.71260 (PMC12441807; doi:10.1002/cam4.71260)
Supplement: Supplementary file 2 — Figure S2: Forest plot of subgroup analyses of progression‐free survival in enrolled patients. [file CAM4-14-e71260-s001.docx]

**Supplementary Information**


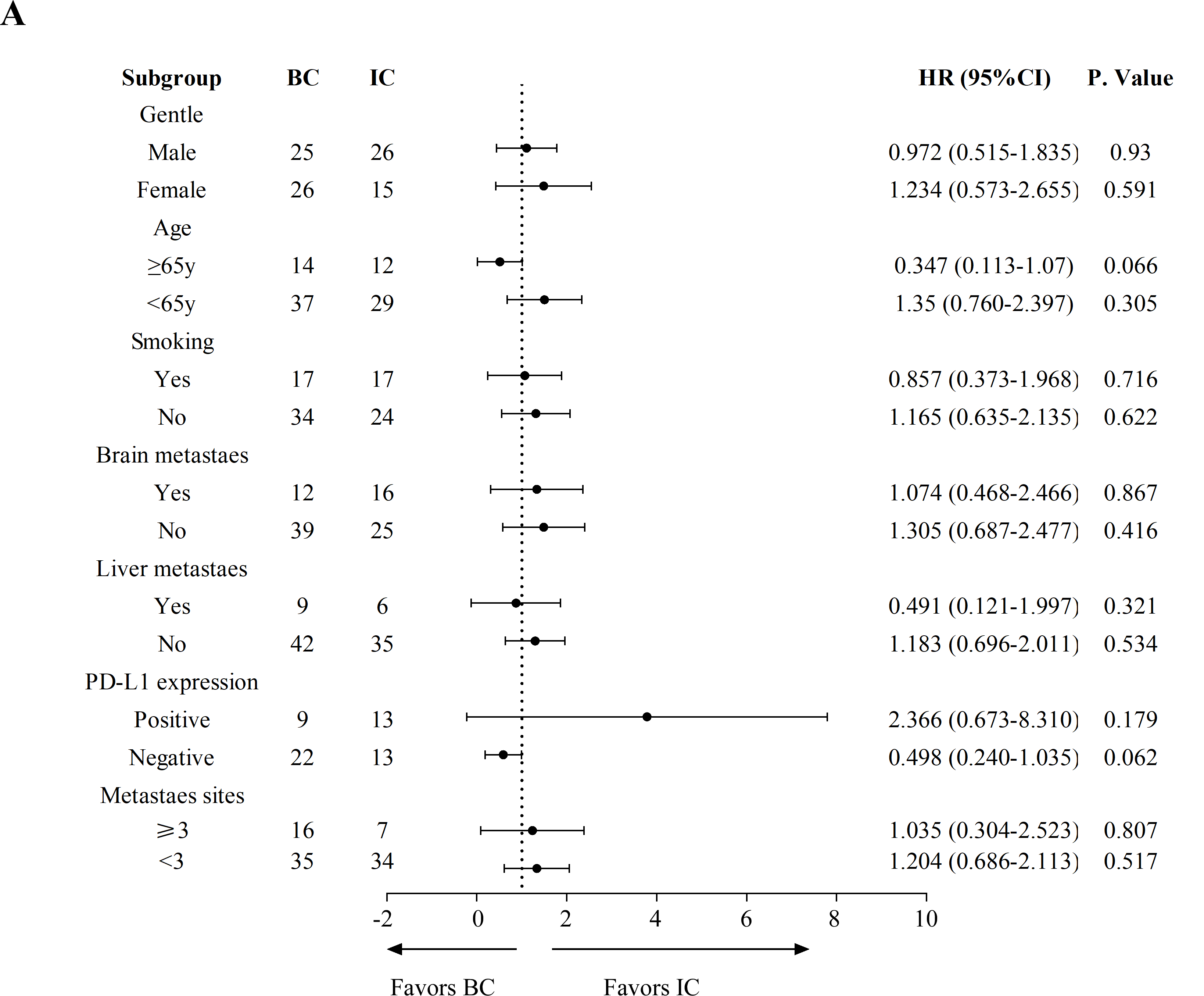


**Figure S2** Forest plot of subgroup analyses of progression-free survival in enrolled patients
